# Polyurethane Degradable Hydrogels Based on Cyclodextrin-Oligocaprolactone Derivatives

**DOI:** 10.3390/gels9090755

**Published:** 2023-09-16

**Authors:** Alexandra-Diana Diaconu, Corina-Lenuta Logigan, Catalina Anisoara Peptu, Constanta Ibanescu, Valeria Harabagiu, Cristian Peptu

**Affiliations:** 1 “Petru Poni” Institute of Macromolecular Chemistry, Aleea Grigore Ghica Voda 41A, 700487 Iasi, Romania; diaconu.diana@icmpp.ro (A.-D.D.); hvaleria@icmpp.ro (V.H.); 2 Department of Natural and Synthetic Polymers, Faculty of Chemical Engineering and Environmental Protection, “Gheorghe Asachi” Technical University of Iasi, 71, Prof. Dr. Docent Dimitrie Mangeron Street, 700050 Iasi, Romania; savincorina@yahoo.com (C.-L.L.); catipeptu@yahoo.co.uk (C.A.P.); cibanescu@tuiasi.ro (C.I.)

**Keywords:** cyclodextrin, oligocaprolactone, isophorone diisocyanate, crosslinking, hydrogel, degradable, polyethylene glycol, levofloxacin, dynamic rheology

## Abstract

Polymer networks based on cyclodextrin and polyethylene glycol were prepared through polyaddition crosslinking using isophorone diisocyanate. The envisaged material properties are the hydrophilic character, specific to PEG and cyclodextrins, and the capacity to encapsulate guest molecules in the cyclodextrin cavity through physical interactions. The cyclodextrin was custom-modified with oligocaprolactone to endow the crosslinked material with a hydrolytically degradable character. SEM, DTG, and FTIR characterization methods have confirmed the morphology and structure of the prepared hydrogels. The influence of the crosslinking reaction feed was investigated through dynamic rheology. Further, thermal water swelling and hydrolytic degradation in basic conditions revealed the connectivity of the polymer network and the particular influence of the cyclodextrin amount in the crosslinking reaction feed on the material properties. Also, levofloxacin was employed as a model drug to investigate the drug loading and release capacity of the prepared hydrogels.

## 1. Introduction

Covalent polymer networks composed of polyethylene glycol (PEG) and cyclodextrins (CD) are particularly interesting for combining the hydrophilicity and flexibility of PEG chains with the capacity of cyclodextrins to encapsulate active principles. These materials’ excellent properties are recommended for applications such as tissue engineering, drug delivery, coatings, solid–solid phase-changing materials, etc. [1,2,3,4,5,6].

The crosslinking strategies involve a wide range of approaches which are most often taking advantage of the common hydroxyl groups available on both components [7]. Among the employed crosslinking agents the isocyanates are attractive due to their availability and the chemical reactivity that allows controlling the crosslinking process. Marrying two components with relatively different solvent interaction properties may be challenging, and using various diisocyanates-cyclodextrins combinations generally resulted in macroscopically homogeneous networks [1,2,3,4,5,6]. However, scanning electron microscopy (SEM) investigations revealed microphase separations [8]. Importantly, polyurethane cyclodextrin-PEG networks were demonstrated through electron spin resonance spectroscopy to retain the cyclodextrin capacity to form host-guest complexes post-polyaddition process [9].

PEG networks were successfully formulated by incorporating both native and modified cyclodextrins. In these formulations, the highest amount of carbohydrate was determined at a 1/4 molar ratio to PEG for polyaddition reactions involving OH functional groups. Further increase in the carbohydrate amount involved the preparation of hexamethylene diisocyanate-modified cyclodextrins and their crosslinking using amino-end-capped PEG. This particular strategy allowed the preparation of crosslinked networks that had up to 2/1 β-cyclodextrin/PEG molar ratio [10].

Among the employed monomers for PEG-CD polyurethane synthesis, such as diphenylmethane diisocyanate (MDI) [4] or hexamethylene diisocyanate (HDMI) [1,2,3,5,6], isophorone diisocyanate (IPDI) represents an important monomer due to its asymmetric reactivity of the aliphatic and cycloaliphatic isocyanate moieties [11]. This difference is particularly important for two-step processes. The prepolymerization step is crucial, and the chain extensions should be avoided. The difference in reactivity between the IPDI isocyanate functions, especially in reactions catalyzed by dibutyltin dilaurate (DBTL), limits the undesired reactions [12]. Usually, polyols are end-capped with IPDI in the first stage followed by a second step that shapes the final material as linear polymers or crosslinked networks. Various characterization techniques are employed for the evaluation of PEG end-capping reaction with IPDI and, lately, MALDI mass spectrometry has emerged as a robust and precise method to monitor the prepolymers [13,14].

The role of cyclodextrin as a polyol in the synthesis of polyurethane networks is defined by the sterically hindered hydroxyl groups as opposed to other polycarbohydrates with a loose structure that allows better accessibility in polyaddition reactions. Thus, once some of the OH groups of the cyclodextrin are involved in the crosslinking process, the neighboring unreacted functions become inaccessible and ultimately remain unreacted [4]. Also, β-CD’s OH groups are characterized by different reactivities in chemical reactions. Thus, OH groups connected to the C6 of the glycoside ring have increased nucleophilicity while those connected to the C2 and C3 are more acidic and interact with their neighbors through hydrogen bonds [15]. Regarding the reactivity towards the isocyanates, the OH groups of secondary alcohols (OH-S) are less reactive than those of primary alcohols (OH-P), as demonstrated by kinetic studies comparing, e.g., the reactivity of n-propanol and isopropanol with IPDI [16]. However, other factors may affect the overall reactivity such as the employed solvent and hydrogen bonding among others [17]. Cyclodextrin-oligolactide oligoesters (CDLA), which are characterized by an increased content of OH-S functions as compared with the native corresponding cyclodextrins, revealed during the dynamic rheology monitoring of the crosslinking with PEG-(NCO)_2_ a slower gelation process associated with diminished system reactivity [14]. Moreover, the insertion of oligoester sequences rendered a biodegradable character to the polyurethane networks proving the involvement of oligolactide OH groups in the crosslinking processes. Therefore, understanding the specific role of native and modified cyclodextrin as crosslinking agents in isocyanate polyaddition reactions raises specific problems that need further elucidation.

The current study is part of our efforts to comprehend the structure-properties correlations in CD-PEG crosslinked networks. The work is focused on the hydrogels produced through a polyaddition reaction between IPDI end-capped PEG chains and custom-prepared β-cyclodextrin-oligocaprolactone (CDCL). Dynamic rheology investigation provides further understanding of how altering the nature of the OH groups and the reaction feed parameters influence the progression of the crosslinking reaction. In addition, we also give insights into how chemical composition impacts network properties, such as swelling behavior, thermal stability, hydrolytic degradability, and drug release profiles.

## 2. Results and Discussion

**Scheme 1:**
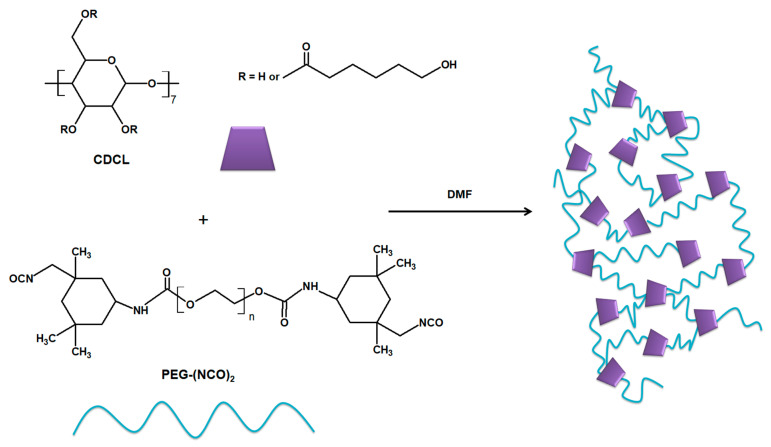
Schematic representation of the crosslinking reaction employed for the synthesis of the CDCL-PEG hydrogels.

Previously, we showed that cyclodextrin derivatives modified with oligolactates mainly at the primary OH groups increase the crosslinking period as compared with the native cyclodextrins [14]. Herein, we aim to further extend the existing knowledge by employing another cyclodextrin derivative modified with oligocaprolactone chains (CDCL), expecting a different crosslinking behavior and, hence, significantly different polymer network properties. The representation of the crosslinking process for the hydrogel preparation is presented in Scheme 1.

### 2.1. Prepolymers

The IPDI functionalized the PEG chains at both ends—PEG-(NCO)_2_ were prepared according to the previous literature [14,15], ensuring a high degree of the terminal OH groups’ transformation into isocyanate functions. The reactivity difference between the isocyanate groups of IPDI in the conditions of DBTL catalysis ensures that the most reactive isocyanates, directly connected to the cyclohexane ring [11], are connected to the PEG.

**Figure 1:**
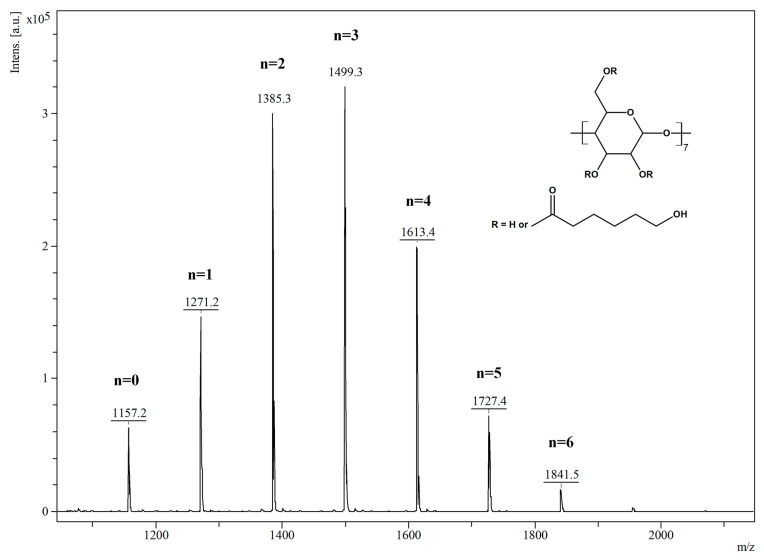
MALDI mass spectrum of the β-cyclodextrin-oligocaprolactone prepolymer (annotated n values represent the number of monomer units attached to the β-cyclodextrin).

The CDCL derivatives were synthesized according to a previously published study [18]. The product characterization by MALDI MS is further discussed. Thus, the structure and the average substitution degree of the CDCL product are obtained based on the MALDI MS spectrum given in Figure 1.

The peak-to-peak difference is 114 which corresponds to the 6-hydroxy-hexanoate constituent unit, annotated as CL. The observed MS peaks may be described as β-cyclodextrin esterified with oligocaprolactones having a variable number of CL units. This interpretation is based on the following equation: *m/z* = 1134 (*β-CD*) + *n* × 114 (*CL*) + 23 (*Na*) where n represents the number of CL units contained by the product associated with a specific peak. For example, the base peak found at *m*/*z* = 1499.3 corresponds to a CDCL product having 3 CL units. Based on the relative intensities of the MS peaks we determined the average degree of substitution of three CL units per cyclodextrin. Nuclear magnetic resonance (NMR) analysis previously described revealed a random substitution at the level of OH groups belonging to either C2, C3, or C6 o β-CD [18]. This substitution pattern of CDCL theoretically ensures the increase of the primary OH group number, which may influence the crosslinking process.

### 2.2. Hydrogel Synthesis

Hydrogels were obtained through a crosslinking reaction between CDCL and IPDI-modified PEG oligomers using N,N-dimethylformamide (DMF) as the crosslinking environment (Scheme 1). The crosslinking reaction was performed for different CDCL/PEG-(NCO)_2_ molar ratios (Table 1) and the final hydrogels were obtained after thorough purification with high yields, thus reflecting an effective polymer network formation through the incorporation of both CDCL and PEG-IPDI oligomers.

It is worth mentioning that replacing cyclodextrin-oligolactide (CDLA) [14] with CDCL in these crosslinking systems improves their capacity to incorporate more cyclic carbohydrates due to the increased reactivity of the latter. Previously, the hydrogel synthesis performed in the presence of CDLA oligoesters, in similar reaction conditions, led to homogeneously crosslinked networks only for a 1/4 molar ratio between CDLA and PEG-(NCO)_2_. In the case of CDCL, we remarked that a 1/2 molar ratio also led to crosslinked networks with good mechanical integrity, which allows the manipulation of the hydrogels during purification procedures. “The CDCL and CDLA oligoesters differ at the level of OH type groups, CDCL presenting more primary OH groups, specifically OH-P type, which is more reactive towards IPDI, as demonstrated through kinetic studies [19]”.

The CDCL and CDLA oligoesters exhibit variations in the types of OH groups they possess, with CDCL having a higher amount of primary OH groups, the OH-P type. These OH-P groups exhibit greater reactivity towards IPDI, as confirmed by kinetic studies [19]. Therefore, we may expect increased crosslinking system reactivity in the presence of CDCL which provides rapid and homogeneous connectivity throughout the network. On the other hand, previously employed CDLA oligoesters, which have more OH-S functions, were unable to produce a continuous network for a 1/2 molar ratio of CDLA/PEG-(NCO)_2_ [14].

**Scheme 2:**
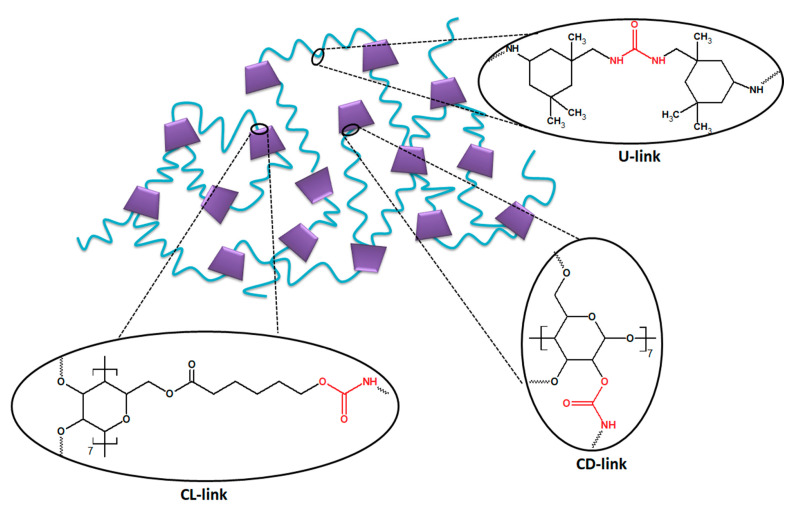
Representation of possible network connections in the CDCL-PEG networks: connections to the cyclodextrin core through oligocaprolactone—CL-link, direct connections to the CD core—CD-link, and urea connections—U-link.

The cyclodextrin grafting with oligocaprolactones, terminated with primary OH groups and having increased mobility related to the flexibility of the ester bonds, leads during the crosslinking process to the formation of CL network bridges, nominated as CL-link, as represented in Scheme 2. In the conditions of increased isocyanate amounts the unmodified OH groups will also contribute to the network connectivity through CD-links, direct connections to the cyclodextrin core. Also, during crosslinking, secondary reactions are possible leading to the formation of U-links, the urea bonds resulting from the water purification of the hydrogels [20].

### 2.3. Hydrogels Morphology and Structure

**Figure 2:**
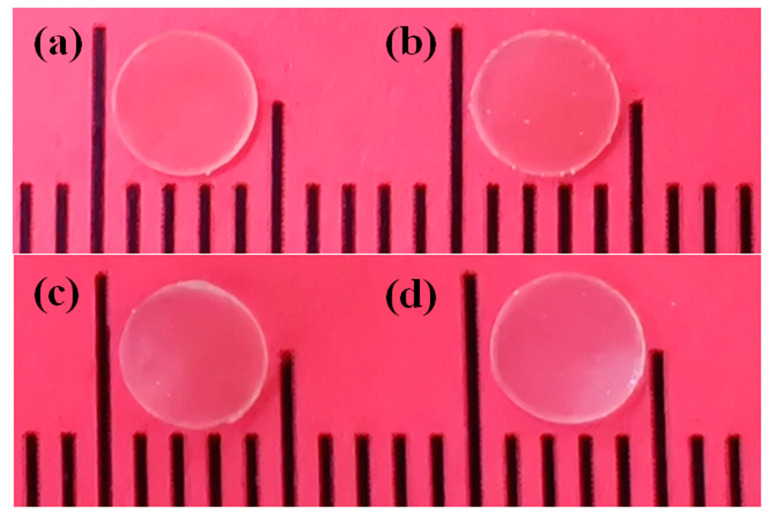
Macroscopic description of the CDCL hydrogels (**a**) C2, (**b**) C4, (**c**) C8, and (**d**) C12.

The CDCL hydrogels were transparent and flexible without noticeable differences at the macroscopic level between C2-C12 syntheses, as observed from the pictures presented in Figure 2.

**Figure 3:**
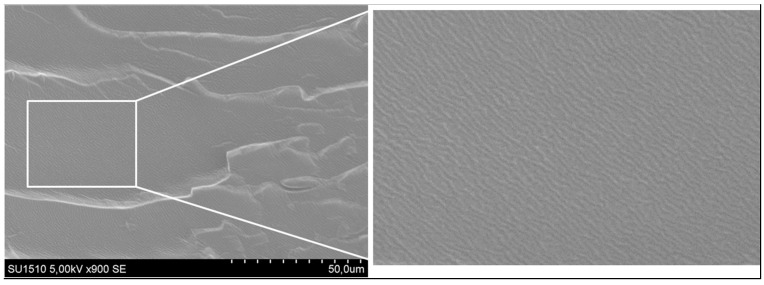
SEM micrograph of C2 at ×900 magnification, cross-section view, and highlight of the phase separation.

The insights into the three-dimensional network and morphology of the hydrogel were derived from the SEM analysis of freeze-fractured hydrogels. The images presented in Figure S1 reveal that at the microscopic level, the prepared hydrogels are homogeneous without microscopic pores or phase separations. Generally, SEM analysis was not able to evidence specific aspects related to the C2–C12 hydrogel formulations that may be connected to the specific amounts of components in the synthesis feed. However, the freeze-fractured image of the C2 hydrogel formulation highlighted in Figure 3 revealed relatively regular wrinkles at the nanometer level that may indicate a specific spatial arrangement between the PEG and CDCL phases and, hence, phase separation. Most likely this phenomenon is related to the increased amount of CDCL in the synthesis feed of the C2 sample, which accentuates the separation between PEG and CDCL-rich phases. Thus, we may consider that during the crosslinking process, the two reactive components were homogeneously distributed throughout the hydrogel volume at the macroscopic level, ensuring the hydrogel connectivity despite different macromolecular architectures: PEG-(NCO)_2_ chains are mobile and flexible while CDCL structures are stiff and bulky. However, based on these results we cannot exclude the formation of separate phases at the nanometric scale, as previously observed in the case of cyclodextrin-polytetramethylene glycol polymer networks [8].

The content and the crosslinking homogeneity may be further evaluated by structural characterization and the determination of the physical–chemical properties. Thus, properties such as porosity, water swelling, mechanical strength, crosslinking density, biodegradability, surface chemistry, and others are interrelated and determine the applicability of the materials in biological fields [21].

**Figure 4:**
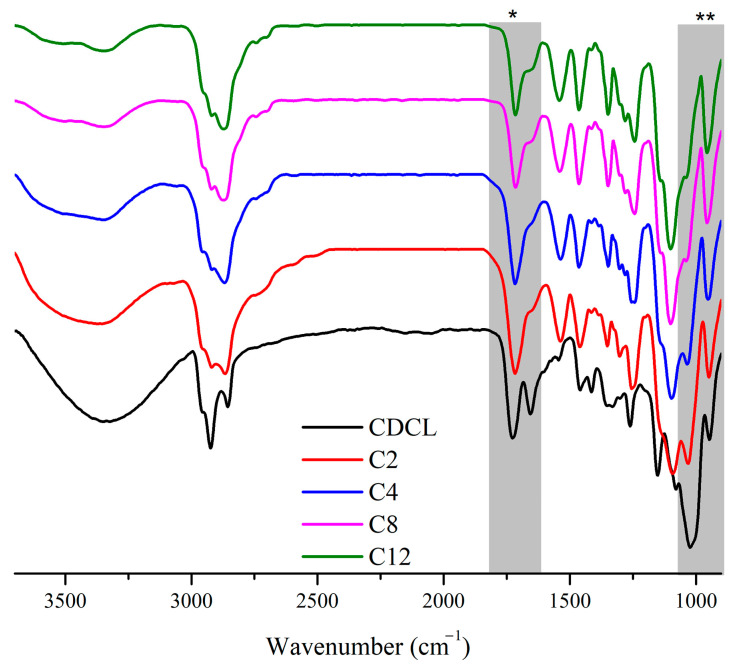
Overlapped FTIR spectra for the structural characterization of the CDCL-PEG hydrogels; highlighted regions—* (carbonyl from urethane and urea bonds) and ** (ether bonds from the carbohydrate).

The result of the crosslinking processes was structurally evaluated using the FTIR spectroscopy analysis, as presented in Figure 4. FTIR spectrum of CDCL is also depicted for the ease of comparison of the chemical environment changes. The adsorption bands at 3348 cm^−1^ and 1655 cm^−1^ are attributed to the O-H stretching vibration and bending vibration of water molecules [22], respectively; the bands at 2924 cm^−1^, 1414 cm^−1^, and 947 cm^−1^ to the vibration of C–H bonds, and those from 1151 cm^−1^, 1080 cm^−1^ and 1022 cm^−1^ are assigned to the stretching vibration of C–O. Moreover, specific bands from CL units can be observed at 1726 cm^−1^ (C=O ester), 1460 cm^−1^ (C-H), and 1261 cm^−1^ (C-O). The presence of these bands confirms the presence of CL chains on the CD molecule, as previously shown for other esterified cyclodextrins [14,23].

In the case of the synthesized hydrogels the specific band of isocyanate groups (2270 cm^−1^) is absent from all spectra and the formation of the urethane connections to cyclodextrin derivatives may be correlated with the specific bands observed at 3370 cm^−1^ (N-H groups), at 1717 cm^−1^ (C=O) and 1537 cm^−1^ (HN–CO). Moreover, specific PEG bands can also be observed at 1460 cm^−1^ (O-H), 1350 cm^−1^ (O-H), 1254 cm^−1^ (C-H), and 1092 cm^−1^ (C-O). The band observed at 1640 cm^−1^ is associated with the carbonyl bond of urea which is formed during the gel purification with water [1,2,3,4,6,14]. This band is more pronounced in the case of hydrogels C8 and C12 which had an excess of isocyanates during synthesis. The observation of urea linkages confirms that although the molar ratio between OH and NCO functions was in favor of the former, the crosslinking process results in insufficient coverage of the NCO functions that remain unreacted with the cyclodextrins and become available for the CO_2_ elimination in the presence of water and further formation of urea links. U-links, together with CD- and CL-links, are completing the 3D structure of the hydrogels. Although the C2 hydrogel has the lowest amount of U-links, their presence in the conditions of excess OH groups reveals that the crosslinking process lacks homogeneity, most probably because of dense spatial distribution and low mobility of the CDCL moieties.

The presence of CL carbonyl groups in the hydrogels, associated with the band that should be observed at 1726 cm^−1^ (C=O ester), is difficult to differentiate because of the overlapping with the band corresponding to the carbonyl bond from polyurethane (1717 cm^−1^), as may be observed from the * highlighted region in Figure 4. However, the presence of CDCL in the hydrogels may be associated with the intensity decrease in the band at 1022 cm^−1^ (the stretching vibration of C–O in carbohydrate moiety), which is fading with decreasing amount of CDCL in the hydrogels, ** highlighted region in Figure 4. The FTIR analysis was in excellent agreement with previous studies [1,2,3,4,14], thus confirming the structural analysis.

**Figure 5:**
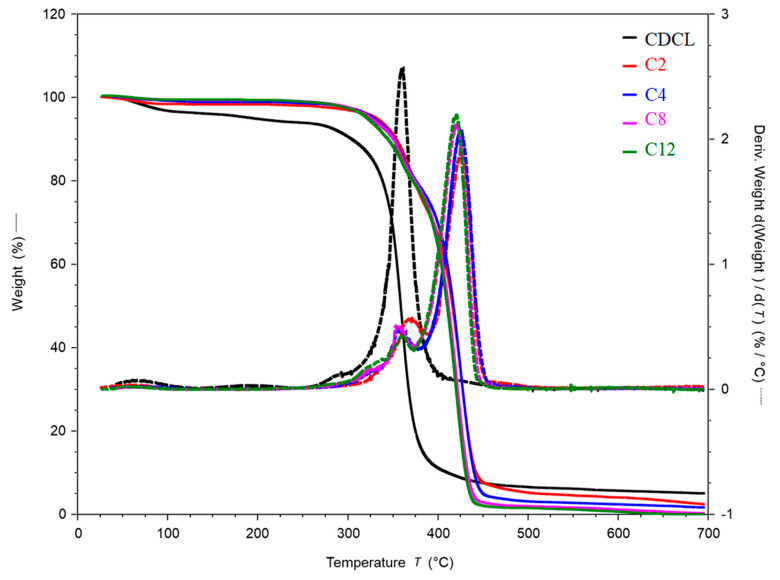
Thermal degradation profiles for the hydrogel samples obtained through TGA—solid lines and DTG—dashed lines.

The hydrogel content was indirectly confirmed through the observation of the thermal degradation process specific to the crosslinked moieties (Figure 5 and Table S1). The thermal degradation curves of the synthesized materials presented in Figure 5 are plotted together with the degradation profile of the CDCL component for comparative evaluation. CDCL presents two stages of degradation, a smaller one, characteristic of short chains of oligocaprolactone, and a considerably larger one, characteristic of oligosaccharide residue [24].

Hydrogels are characterized by three stages of characteristic thermal degradation: urethane bonds followed by the concomitant degradation of oligosaccharides partially overlapped with the cleavage of linkages polyurethane and PEG residues (Table S1). The region between 300 and 340 °C shows degradation profiles in agreement with the preponderance of urethane bonds in C2–C12 samples [25]. Also, the influence of the increasing carbohydrate amount, from C12 to C2, is apparent in the region situated between 340 and 400 °C. The final and most predominant degradation stage is associated with the PEG chains. The amount of CL oligoester units, expected to appear before 300 °C, is likely insufficient to significantly impact the degradation profile.

### 2.4. Dynamic Rheology Investigation of the Crosslinking Reactions

**Figure 6:**
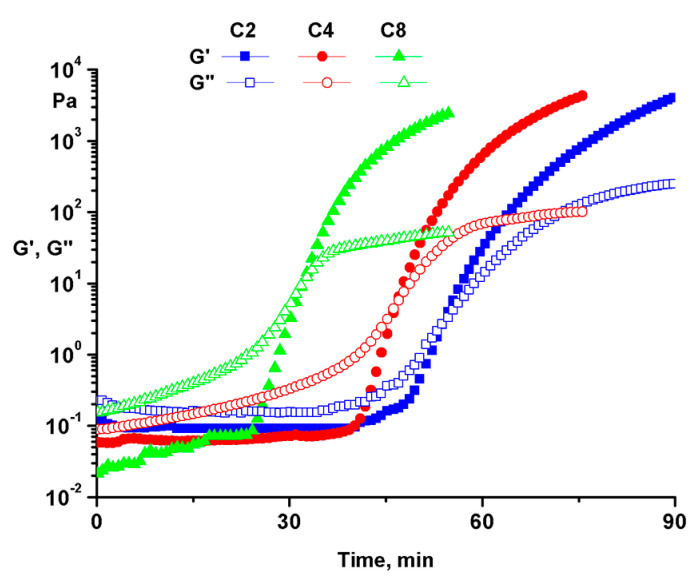
Comparative time sweep rheological test of the CDCL-PEG crosslinking systems for different CDCL/PEG molar ratios (C2—1/2, C4—1/4, C8—1/8).

The characteristic gelation behavior through dynamic rheological measurements was employed to better understand the effect of the cyclodextrin derivatization with oligocaprolactone on the crosslinking reaction. The measurements of the specific variation of the storage modulus (G′) and loss modulus (G″), respectively, for each reaction system, revealed the onset of gelation, defined as the crossover point of the viscoelastic moduli. In the beginning, the reaction system is in a liquid state with G″ > G′ but, after a specific period, the polyaddition reaction leads to a steep increase in G′, accompanied by a moderate increase in G″. This evolution of the viscoelastic moduli leads to a crossover moment when G′ = G″, denoted as the gelation time (T_x_, where x nominates the hydrogel sample) [26]. Comparative time sweep rheological tests for three different CDCL/PEG-(NCO)_2_ molar ratios in the reaction mixtures are presented in Figure 6. The measured G′/G″ crossover characteristic time values (Table S2) are increasing with the decrease in the cyclodextrin relative amount in the feed, in the order T_C2_ > T_C4_ > T_C8_. This behavior was also observed in our previous study for similar crosslinking systems containing β-CD and CDLA instead of CDCL [14] and was explained through the diminished availability of the isocyanate moieties. The critical step in the gelation process is the development of connections between neighboring cyclodextrins and this process is highly influenced by the concentration of OH functions which are less mobile and heterogeneously reactive. Thus, for high OH group content, such as the C2 reaction system, the appearance of network bridged connections between different cyclodextrins takes a longer time, as noticed in our time sweep experiments. As the OH coverage by the NCO functions improves, from C2 to C8 homolog series, the network connections are developing faster, thus explaining the observed differences in the gelation behavior.

**Figure 7:**
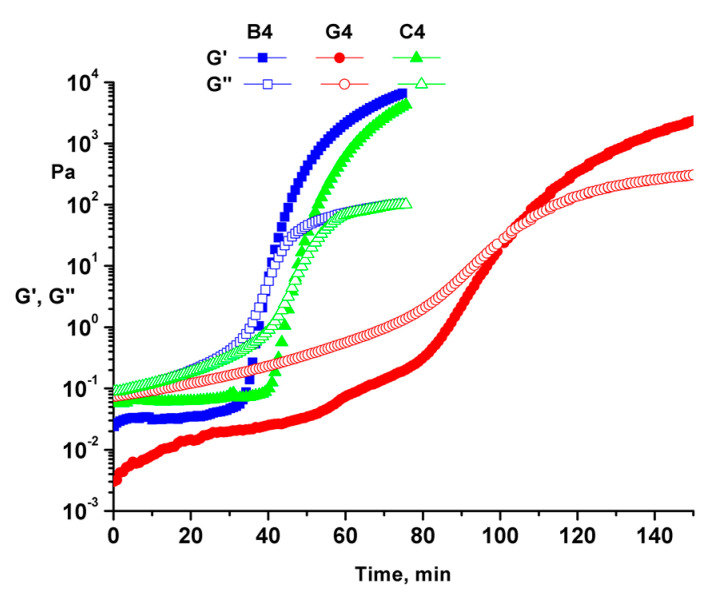
Comparative gelation profiles observed through dynamic rheology for the crosslinking processes conducted in the presence of different cyclodextrin derivatives: β-cyclodextrin (B4*), β-cyclodextrin-oligolactide (G4*), and β-cyclodextrin-oligocaprolactone (C4) samples (*—previously published [14]).

The main question that should be answered is related to the effect of β-CD modification with CL moieties on the course of the crosslinking reaction. The comparative rheological evaluation of three systems (CD-B4, CDLA-G4, and CDCL-C4) having the same molar ratio cyclodextrin derivative vs. PEG-(NCO)_2_ chains was also employed to assess the effect of the CDCL in the crosslinking reaction. The B4 represents the hydrogel obtained using native β-CD and the G4 is obtained using CDLA [14]. The gelation profile of the C4 system plotted together with that of G4 and B4 (Figure 7), revealed that the T_C4_ is slightly higher than T_B4_ and significantly smaller than T_G4_.

The reaction systems showed significantly different crosslinking behavior associated with the crosslinking agents (CD, CDLA, and CDCL) and ultimately associated with the structural differences related to the particular nature of the OH groups. Thus, in the case of CDLA derivatives the balance of OH groups is shifted in favor of OH-S, which led to an increase in the gelation time as compared with the gels crosslinked with CD. Herein, the derivatization of the CD with oligocaprolactone at best preserved, if not improved, the primary OH groups (OH-P) content. Theoretically, random esterification of the cyclodextrin leads to a decrease in the total number of the OH-S groups. NMR characterization revealed that for the conditions employed to synthesize CDCL derivatives, the substitution occurs predominantly at the secondary OH groups [18]. Therefore, we may expect the substitution influence over the crosslinking speed to be significant. However, we noticed that the T_C4_ value was slightly higher than T_B4_, signifying a relatively similar reactivity for both CDCL and CD reaction systems. Besides the influence of the OH type, the steric hindrance exerted by the oligocaprolactone chains over the unsubstituted OH groups may explain the slightly longer gelation duration observed for CDCL.

The rheological measurements allowed us to investigate the macroscopic properties of the hydrogels swollen in DMF, obtained during the time sweep experiments described above. The CDCL-PEG materials have rheological characteristics of a hydrogel with a constant G′ value that is greater than G″ over the entire strain range of 0.01 ÷ 10% (Figure S2).

The variable amount of CDCL in the crosslinking feed influences the rheological parameters revealing higher values of the differences between dynamic moduli G′ and G″, but the limits of the linear viscoelastic range is 5% for all tested samples (Table S3). Thus, the hydrogels exhibited solid-like behavior where the storage modulus (G′) is larger than the loss modulus (G″). Frequency sweep tests emphasized the elastic response of the hydrogels, which is slightly dependent on frequency over the entire range of 0.1 ÷ 500 1/s (Figure S3). The cohesion resulting from the development of the urethane bridges throughout the polymer network is highlighted by the high values of dynamic moduli (G′ and G″).

### 2.5. Thermal Properties

**Figure 8:**
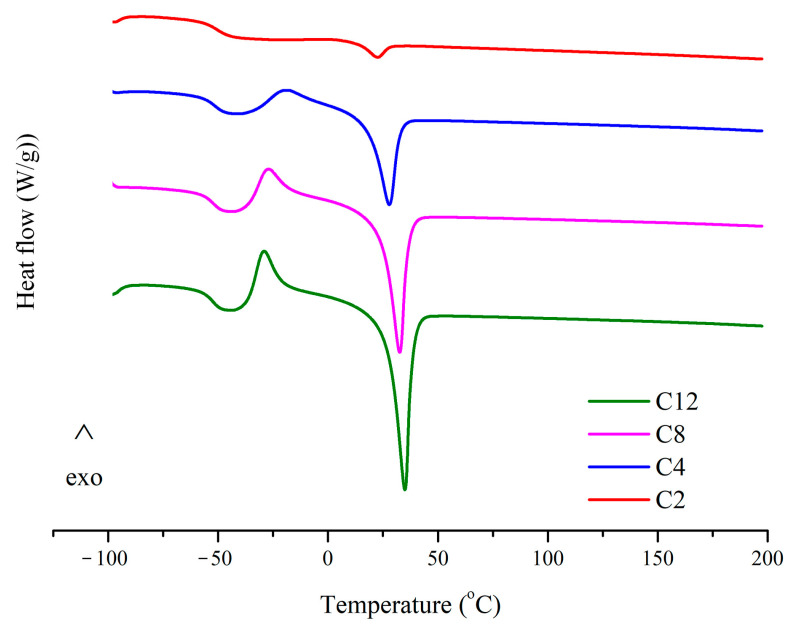
Differential scanning calorimetry analysis of CDCL-PEG hydrogels.

DSC analysis was employed for the delineation of the structure-thermal properties relationship. From Figure 8 we may observe that all the hydrogels present a glass transition associated with the PEG chains (exact values are given in Table S4). Further, hydrogels undergo an exothermic transition, only in the case of the C4–C12 samples, followed by an endothermic transition.

The exothermic transition is related to rearrangements of the network components by cold crystallization of the amorphous domains. We may notice that this specific transition becomes more pronounced as the PEG ratio in the CDCL-PEG hydrogels increases, increasing the network mobility. The endothermic transition observed for all the hydrogels also follows the increasing PEG content. This transition is associated with a solid-solid phase melting generally noticed for PEG crosslinked networks. This particular process has been associated with the capacity of CD-PEG materials for energy storage [4].

### 2.6. Hydrolytic Degradation

The hydrolytic degradation potential of the prepared hydrogels was investigated using an accelerated process in a basic environment. The chosen degradation conditions are expected to expose the influence of the oligocaprolactone component in the network connectivity and, ultimately, the degradable character of the synthesized material.

**Figure 9:**
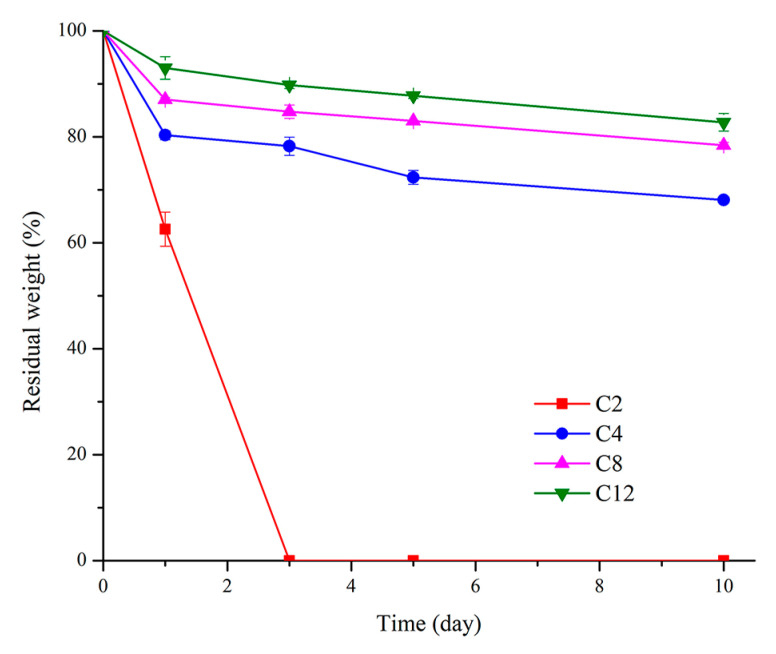
Graphical illustration of the mass loss profiles of C2, C4, C8, and C12 during hydrolytic degradation in basic environment.

The weight loss measurements of the CDCL-PEG series exhibited comparable degradation profiles, as shown in Figure 9. The C4–C12 hydrogels displayed a continuous weight reduction, correlated with the CDCL content present in the system. In the case of the C2 sample which contains the highest amount of CDCL, after 1 day of degradation, the residual weight was almost 60 % and after 3 days the hydrogel was completely disintegrated. However, the C4, C8, and C12 samples showed a continuous mass loss throughout the investigated period, reaching after 10 days residual weight values of 68, 78, and 83 wt%, respectively. The hydrogel degradation in basic conditions involves the cleavage of CL-link bridges. The mass losses observed from this experiment are much higher than the amounts of CDCL present in the initial reaction mixture (as may be observed in Table S5), confirming CL-link connectivity.

Therefore, one may expect the degradability to be dependent on the oligocaprolactone content in each sample. By correlating the gravimetric degradation profiles with the sample’s theoretical oligocaprolactone content, as given in Table S5, it is apparent that a higher content of CDCL leads to more pronounced weight loss. However, the ratio between the CL-link and the other network connectivities may also influence the degradation behavior. Thus, as we showed earlier in Scheme 2, the excess in the synthesis of PEG-(NCO)_2_ chains vs. CDCL moieties (from C2 to C12) leads to the formation of two types of connectivities in the polymer network, direct connection to the OH groups of the cyclodextrin (CD-link) and connections through the terminal CL OH groups (CL-link). We may infer that, in the case of the C2 sample, the rapid disintegration signifies that the majority of the network connections are through CL-link. MS characterization revealed that each cyclodextrin has an average of 3 CL chains and, in consequence, the reaction feed has a molar ratio between OH groups belonging to CL and PEG-(NCO)_2_ chains of 3 to 2. By increasing the amount of PEG-(NCO)_2_ we may observe that, in the case of C4, this ratio is reversed to 3/4, and as the series continues, the isocyanate groups exceed the OH of CL chains. Thus, moving from C4 to C12 the number of CD-links is becoming predominant, which leads to diminished weight loss through degradations. Nevertheless, even if the cleavage of ester bonds does not produce weight loss, due to complementary connectivity, we may expect effects on the network continuity affecting the microscopical aspect.

**Figure 10:**
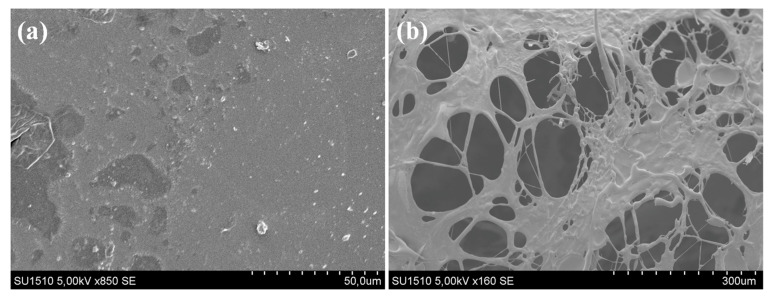
Surface SEM micrographs of C2 sample: (**a**) initial and (**b**) after 1 day of hydrolytic degradation in a basic environment.

Therefore, the modifications of the hydrogels aspect during the degradation period were further investigated through SEM (Figure 10). Before degradation, the sample’s surface is straight with some rugosity (see Figure 10a). However, after a brief period of 24 h (Figure 10b), the sample undergoes significant disintegration associated with a remarkable weight loss and large pores emerge. The hydrolytic degradation of the C2 sample caused a complete transformation of the material, from a flexible, colorless gel to a delicate, thin, and difficult-to-handle material. Further degradation led to the complete dissolution of C2 in the water environment.

**Figure 11:**
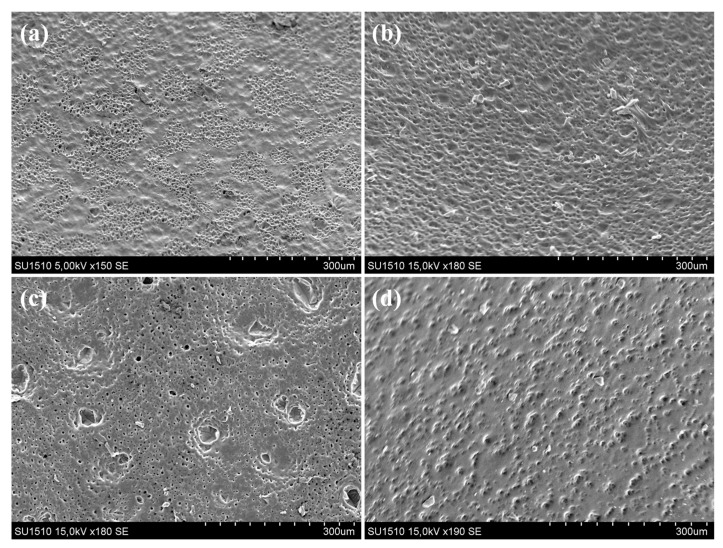
Surface micrographs of the C4 sample after (**a**) 1 day, (**b**) 3 days, (**c**) 5 days, and (**d**) after 10 days of degradation.

The C4 sample degradation occurs on the surface, as appears from the SEM micrographs captured in the cross-section (Figure S4). Notably, there are no significant changes observed in the sample section, and no pores are detected that could have resulted from the degradation process. However, the surface suffers increasing degradation signaled by the formation of pores with increasing size as the degradation period is extended up to five days (Figure 11).

The images obtained after 10 days of degradation revealed the disappearance of the pores and the formation of protuberances (Figure 11d). We may consider that the porous layer has been eroded and the remaining surface material has exposed microphases that are erosion-resistant as compared with the surrounding matrix. Surface erosion would be explained by a water diffusion gradient favoring surface water accumulation, as observed in the case of hydrophobic samples [27,28]. Water swelling experiments revealed that all the hydrogels, including C4, swell homogeneously without macroscopic discontinuities suggesting a relatively equal water repartition in the sample volume. Thus, a preferential surface water swelling may be disregarded.

The structural incompatibility between PEG chains and CDCL oligosaccharide, together with the positioning of the IPDI moieties in the vicinity of the CDCL network nodes, may induce certain hydrophobicity around cyclodextrins and finally lead to the formation of CDCL-IPDI rich phases surrounded by PEG rich hydrophilic phases. Considering the theoretical content of the C4 hydrogel we may observe that the CDCL motifs are almost 25% wt. from the total content, justifying the description of CDCL-rich microphases dispersed in a PEG matrix. The size of the CDCL microphase clusters may be derived from the SEM micrograph (Figure 11), being situated between 5 and 10 μm. On the other hand, these CDCL-rich regions are the degradable part of the hydrogel and it appears that the overall degradation of C4 is influenced by the cluster’s exposure starting from the surface.

Also, in our attempt to explain the morphological alterations throughout degradation, we should consider the joint network connectivity through CL-link, CD-link, and U-link. In the case of the C2 sample, where full disintegration was noticed after 3 days, CL-links are predominant. However, for the C4 sample, the CL-link/(CD-link + U-link) ratio (degradable/non-degradable connections) decreases and, consequently, the necessary time to disintegrate the network increases. Moreover, the molar ratio between the NaOH and the CDCL, employed in the experiments, matches the OH functions content in the tested hydrogel sample, having the same order of magnitude (molar ratio CDCL/NaOH ~ 10^−5^/10^−5^). Therefore, the hydrolytic agent may be trapped by the surface unreacted OH groups from the network, thus eroding only a thin layer, as observed for other systems [28].

C8 sample also presents a surface erosion (Figure S5), but the lower content of CDCL in the initial feed leads to a faster crosslinking, as revealed by dynamic rheology, and hence to smaller hydrophobic microphases (Figure S5b). The presence of CDCL clusters is less obvious and the degraded surface presents more wrinkles than pores (Figure S5c,d). On the other hand, because of the higher molar ratio of isocyanates vs. CDCL, more CD-link and U-link bridges are formed and, consequently, the network becomes more hydrolytically resistant. Therefore, upon the cleavage of ester CL-link bridges, instead of weight loss due to complete detachment of the CD or PEG residues, we notice the crosslinking alteration and surface tensions with wrinkles formation, as becomes obvious from the SEM micrographs taken after 10 days of degradation (Figure S5d).

Cros-section SEM micrographs also confirm network alteration (Figure S6c), the sample becomes more affected, and small cracks start to appear as a consequence of the fracturing process during sample preparation for SEM investigation. Also, because of the lower CDCL content, the diffusion of the NaOH is deeper and affects a thicker sample layer.

**Figure 12:**
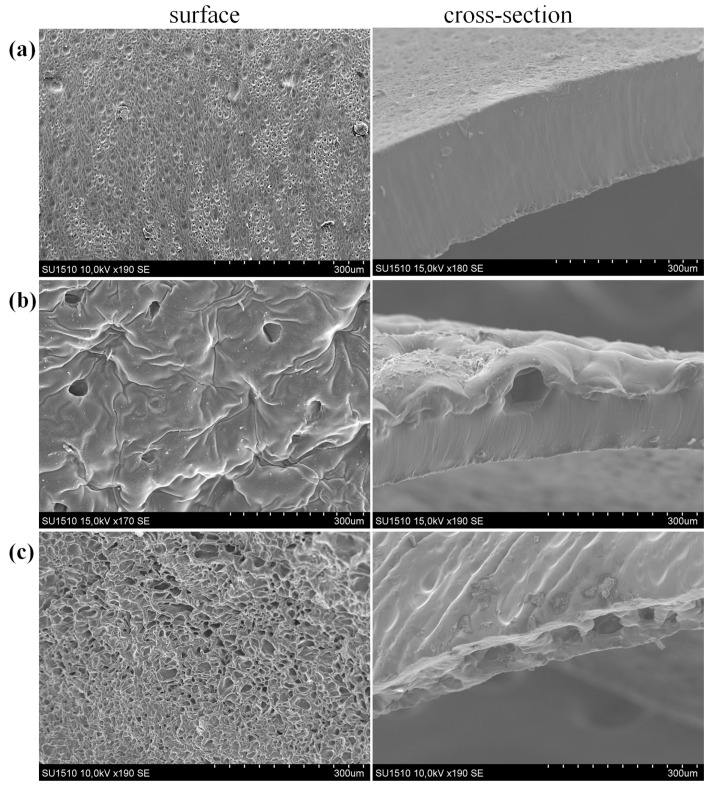
SEM micrographs of the C12 sample after (**a**) 1 day, (**b**) 3 days, and (**c**) after 10 days of degradation.

The C12 sample has even less CDCL content and degrades rapidly due to increased network homogeneity and improved exposure to the basic environment. The effect on the weight loss after 10 days is minimal due to the predominance of the CD- and U-links but the overall structure is the most affected from the analyzed samples series. The morphology analysis reveals that after one day the sample presents surface erosion (Figure 12a—surface and cross-section). Large pores appear after three days of degradation, mostly at the surface but also in the sample volume, as confirmed also by the section images (Figure 12b). After 10 days the depth of the pores increases and the macroscopic aspect is also affected as the C12 sample becomes flattened at the expense of decreased thickness (Figure 12c). This radical network deformation indicates a deep cleavage of the CL-links which is well correlated with a more effective coverage of ester bonds dissociation by the hydrolytic agent.

### 2.7. Water Swelling and Levofloxacin Release

**Figure 13:**
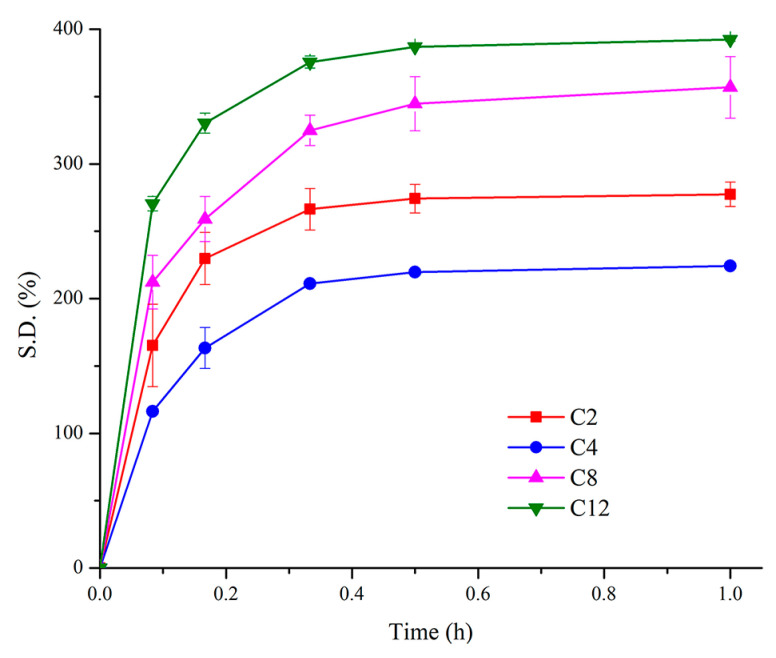
Water uptake behavior of the CDCL-PEG hydrogels.

Morphology, pore size, swelling behavior, and degradability control the structural and bulk properties of the polymer hydrogel. All these properties have an important role in the mass transfer of water and drugs into and out of the hydrogel and thus affect the release kinetics of the incorporated water-soluble drugs. In general, drug release from polymers depends on the overall effect of various factors including water and drug diffusion through the polymer matrix, drug dissolution, polymer swelling, and polymer degradation. Therefore, in the first approach, the water interactions of the CDCL-PEG hydrogels were investigated (Figure 13).

Our data interpretation correlates the swelling behavior, in terms of maximal water uptake, with the crosslinking density given by the amount of the cyclodextrin component and also the PEG content in the respective hydrogel formulation (Table S5).

Generally, the analyzed hydrogel formulations presented a water swelling behavior highly influenced by their hydrophilic character given by the PEG content. Thus, samples C4 to C12 presented an increasing swelling degree (SD) from 200% to almost 400%. This increase may be directly correlated to the increasing amount of PEG in the network composition.

The C2 sample represents an exception from the observed general tendency. The crosslinking density, which depends on the amount of polyol (CDCL), also influences the water uptake. In principle, when the amount of cyclodextrin increases the network density is higher and the water uptake should be lower, as observed for C4–C12 samples. However, we noticed that C2, which is more densely packed due to the highest amount of CDCL component, has a higher water uptake than C4 which has less CDCL, hence a looser network. This is opposed to the generally observed trend. The influence of the PEG content difference between C2 and C4 is rather limited in this situation.

We may infer that a specific network repartition of CDCL moieties for C2 leads to increased SD as compared with C4. DSC confirmed the network rigidity as for C2 the rearrangement of crystalline domains was not observed. Also, the DSC analysis revealed that the melting transition, associated usually with the mobility of PEG chains, is less significant as compared with the other homologs from the CD-PEG hydrogels series, hence signifying that the C2 network is tight.

On the other hand, the increased amount of CDCL during the synthesis of C2 leads to a more homogeneous repartition of PEG component throughout the network. Therefore, we may consider that the PEG chains are more effectively involved in the network formation and create inter-CDCL interstitial spaces that may accommodate water molecules. Moreover, CDCL moieties are less substituted with the hydrophobic IPDI, the OH groups of CDCL are less mobilized into the formation of network bridges and have increased availability for water interactions.

The potential of CDCL-PEG hydrogels as drug delivery systems was investigated by evaluating their drug loading and release efficiency. Levofloxacin (LEV) is a broad-spectrum antibiotic that is active against both Gram-positive and Gram-negative bacteria and used to treat infections including respiratory tract infections, urinary tract infections, endocarditis, meningitis, and pelvic inflammatory disease and is commercially available mostly in oral or intravenous formulations [29]. All pre-weighted formulations (approximately 30 mg) were loaded with LEV solution (20 mg/mL) for 24 h and subsequently, the release ability was determined in phosphate buffer solution (PBS). The amount of LEV loaded in hydrogels (Table S6) was between 8.33 (sample C4) and 20.77 mg (sample C12).

**Figure 14:**
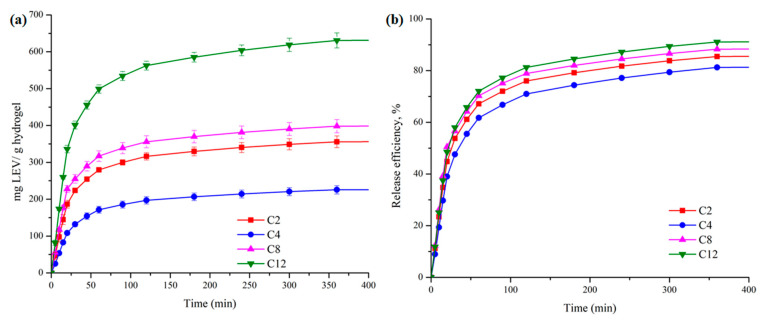
The drug release profiles of the hydrogels: (**a**) the levofloxacin released amounts and (**b**) the release efficiency.

The loading and release results were found in agreement with the results noticed for the water uptake. Increasing the amount of PEG in hydrogel compositions determined the increase in the loading and releasing ability of hydrogels due to enhanced hydrophilicity (Figure 14—highlights the release profiles up to 400 min and Figure S7—presents the release profiles up to 1440 min). The exception is represented by sample C2 which is characterized by a higher loading and release than C4 and this could be explained by the fact that the LEV diffusion and uptake in the polymer network is correlated with the hydrophile character tendency, as noticed during water swelling experiments. The burst release, noticed in the first 50 min (up to 60% of loaded drug), is followed by a sustained release for the next few hours. The delayed release of LEV molecules may be the result of physical interactions with the CDCL cavities.

From loading and release data, LEV release efficiency was determined (Figure 14b). The release efficiency is situated between 81% (C4 sample) and 91% (sample C12). Even though the variation range of release efficiency is only 10%, the efficiency is increasing in the same order C4, C2, C8, and C12 as for swelling degree behavior. Overall, we may consider that LEV’s distribution in the hydrogel network is highly influenced by its water solubility.

Linear plotting of release data, according to the Korsmeyer-Peppas model [30], (Figure S8) shows that the diffusional exponent values are higher than 1 (ranging from 1.35 to 1.48) which means that the mechanism of drug release relates to Super Case-II transport type. For this release mechanism, the drug molecules are entrapped within the hydrogel matrix and released through a combination of swelling, relaxation, and diffusion processes most probably fueled by the increased mobility of the PEG chains fixed at both ends by the cyclodextrins. The erosion of the polymer matrix may be also considered [31], especially when the matrix presents a limited swelling degree. However, in the case of CDCL-PEG networks the degradation at neutral pH did not occur in the timeframe of drug loading and release experiments. Therefore, the erosion contribution to the increase in the diffusional exponent values may be excluded.

## 3. Conclusions

The synthetic polyaddition procedure enabled the preparation of a polyurethane hydrogel composed of cyclodextrin-oligoester-polyethylene glycol, which possesses hydrolytic degradability due to the incorporation of oligocaprolactone sequences. FTIR structural characterization validated the hypothesis over the network connectivity and showed that, besides the urethanes, urea bonds are also formed. The dynamic rheology provided further insights showing that the insertion of caprolactone units on the cyclodextrin does not significantly decrease the overall system reactivity as previously observed in the case of oligolactide-modified cyclodextrins. This effect was associated with the nature of OH groups and provides the possibility to increase the amounts of incorporated carbohydrates in the polymer network through hydroxyl-isocyanate reactions. DSC characterization revealed crystallization and melting processes associated with the network mobility given by the PEG chains. Network connectivity was further delineated by studying hydrolytic degradation. The degradation gravimetric analysis showed that the weight loss depends on the ester linkages. An unexpected opposite trend was remarked when inspecting the physical changes of the homolog series of hydrogels. In moderately basic conditions, the sample containing more CDCL completely disintegrated while other samples showed surface erosion. SEM evidenced more significant physical alteration such as large pores formations and sample flattening for the samples with lower CDCL content, showing the importance of ester bridges in the network connectivity. The potential of the hydrogels prepared in this study as drug-delivery devices for topical applications was demonstrated by conducting in vitro release experiments using levofloxacin as a model drug. The observed mechanism of drug release is determined as Super Case-II transport, associated with the increased mobility of the PEG chains in the polymer network.

## 4. Materials and Methods

### 4.1. Materials

β-cyclodextrin (CD—Cyclolab, Budapest, Hungary) was dried under vacuum (0.01 mbar at 80 °C for 72 h) and kept under an argon atmosphere. ε-Caprolactone (CL—Sigma-Aldrich, Saint Louis, MO, USA), N,N-Dimethylformamide (DMF—Sigma-Aldrich, Saint Louis, MO, USA), and dimethyl sulfoxide (DMSO—Sigma-Aldrich, Saint Louis, MO, USA) were distilled under reduced pressure before use. Polyethylene glycol (PEG, Mn = 2000 g/mol—Sigma Aldrich, Saint Louis, MO, USA) was dried under reduced pressure for 3 h, at 100 °C to remove traces of water. Isophorone diisocyanate (IPDI), dibutyltin dilaurate (DBTL), 4-dimethylaminopyridine (DMAP), Amberlyst 15 hydrogen, and levofloxacin (LEV) were purchased from Sigma-Aldrich, Saint Louis, MO, USA, and were used as received.

### 4.2. Synthesis of Cyclodextrin-Oligocaprolactone—CDCL Derivatives

The synthesis was carried out according to a procedure presented in the literature [17]. DMSO and β-cyclodextrin were placed in a round bottom flask and CL monomer was added in a concentration of 1.8 M, using a molar ratio of 1/8 (CD/CL). The DMAP catalyst was added in a molar ratio of 1/1 to CD. The round bottom flask was kept for 72 h in an oil bath with magnetic stirring and heating at 120 °C. The reaction mixture was mixed with Amberlyst and left under stirring for 2 h to remove the DMAP catalyst followed by dilution in methanol, precipitating it in cold diethyl ether and dried in a vacuum oven (20 mbar) for 12 h at 50 °C. The cyclodextrin-oligocaprolactone product (CDCL) was obtained with a yield of 62% and structurally characterized by MALDI MS and FTIR.

### 4.3. Hydrogel Synthesis

The synthesis of hydrogels was performed through the crosslinking reaction between the OH groups of CDCL and isocyanates groups of bifunctional polyethylene glycol, PEG-(NCO)_2_. First, the PEG-(NCO)_2_ was prepared as previously reported [16,18]: 3 g of freshly dried PEG and 6 mL of dry DMF were added under an Ar atmosphere in a round-bottomed flask equipped with a magnetic stirrer together with 0.66 g (0.62 mL) of IPDI in a molar ratio of PEG:IPDI = 1:2, and one drop (11 mg) of DBTL. The reaction mixture was magnetically stirred for 30 min at 50 °C and mixed with different CDCL solutions in DMF to match the intended CDCL/PEG-(NCO)_2_ molar ratios (*vide infra* Table 1) while keeping the total concentration of 34% (wt.). The reactive mixtures were further homogenized for 10 min at room temperature under magnetic stirring and transferred to a glass vessel in an oven, under Ar protection. Subsequently, the samples were cured at 50 °C for 24 h to form hydrogels. The obtained hydrogels were washed with acetone for 24 h, and with double-distilled water for 24 h (changed twice) to remove the DMF solvent and the unreacted compounds. The water-swollen hydrogels were frozen at −18 °C, lyophilized, and structurally characterized.

### 4.4. Characterization Methods

#### 4.4.1. MALDI MS

Mass spectrometry measurements were performed using the RapifleX TOF-TOF instrument (Bruker, Bremen, Germany). The mass spectrometer was operated using FlexControl 4.0 and the acquired spectra were processed with the FlexAnalysis 4.0 software. The ionization laser power was adjusted to just above the threshold to produce charged species. A total of 18,000 spectra were averaged for each sample analysis.

CDCL analysis was performed as follows: 5 mg of substance was dissolved in 1 mL methanol and mixed using a Vortex Genie-2. The 2,5-dihydroxybenzoic acid (DHB) matrix and NaI solutions were prepared in methanol at concentrations of 20 mg/mL and 5 mg/mL. The method used to prepare the samples was dried-droplet: 20 μL of DHB was mixed with 2 μL of NaI and 2 μL of sample solution. From the mixture, 1 μL was deposited on the MALDI steel plate and left to dry in air at room temperature.

The obtained MALDI MS spectrum was used to calculate the numerical average molecular mass (M_n_) as follows:

(1) $$M_{n}=\frac{\sum \frac{m_{i}}{z}a_{i}}{\sum a_{i}}$$

where m_i_/z is the mass corresponding to the MS peak i and a_i_ represents the intensity of the MS peak i in the mass spectrum.

#### 4.4.2. FTIR

The FTIR spectra were registered in an attenuated total reflection in the range of 600–4000 cm^−1^, at room temperature with a 4 cm^−1^ resolution and by accumulating 32 scans using a Bruker Vertex 70 FTIR spectrophotometer equipped with a ZnSe crystal.

#### 4.4.3. SEM

The morphologies of the dried hydrogels were recorded using the HITACHI SU 1510 (Hitachi SU-1510, Hitachi Company, Tokyo, Japan) Scanning Electron Microscope. The samples were fixed on an aluminum stub covered with a double adhesive carbon band and were placed on the chamber pedestal of a Cressington 108 Sputter Coater device and coated with a 7 nm thick gold layer, under vacuum. Fracture images were obtained on the hydrogel samples’ cross section after rapid freezing in liquid nitrogen using a pair of tweezers.

#### 4.4.4. TGA

The thermal stability of the samples was analyzed using the Discovery TGA 2500 equipment from TA Instruments (New Castle, DE, USA) and the TRIOS software (v5.1.1.46572) was employed for analysis. Thermal degradation analysis was performed under a nitrogen atmosphere and at a heating rate of 20 °C/min.

#### 4.4.5. DSC

The DSC measurements were performed with a heating/cooling rate of 10 °C/min, under a nitrogen atmosphere using a Discovery DSC 250 (TA Instruments). A heating-cooling-heating cycle was performed. The obtained curves were processed with the TRIOS software.

#### 4.4.6. Rheological Measurements

Oscillatory rheological tests were carried out using a Physica MCR 501 (Anton Paar, Graz, Austria) modular rheometer equipped with a Peltier temperature control device. The measurements were performed using 50 mm diameter parallel plate geometry with serrated plates to avoid slippage of the sample. The rheology of the hydrogels was performed at a constant temperature (50 °C) and was studied using oscillatory measurements for the time sweep test (shear strain 5%, angular frequency 10 rad/s).

The hydrogels as resulted from the time sweep experiments were studied using oscillatory rheological measurements: amplitude sweep (shear strain 0.01–10%, angular frequency 10 rad/s), frequency sweep (shear strain 5 %, angular frequency 0.1–500 rad/s). All the experiments were performed at constant temperature (50 °C).

#### 4.4.7. Swelling Degree

The water-swelling behavior of the hydrogel was studied by a gravimetric procedure. Hydrogel samples (40–50 mg) were introduced into glass vials with 3 mL of distilled water and the swollen hydrogels were removed from the vials, gently pressed between two pieces of filter paper to remove any excess water on the surface, and weighed. The weight was monitored until the equilibrium was reached. The swelling degree (S.D.) is defined as:

S.D. (%) = (m − m_0_)/m_0_ × 100 (2)

where m and m_0_ are the swollen mass and the initial (dry) mass, respectively. Each sample was measured in triplicate.

### 4.5. Hydrolysis Degradation of the Hydrogels

The hydrogel samples (30–40 mg) were placed in glass bottles with a 5 mL solution of NaOH 0.01M (pH = 11.77) at a constant temperature (37.5 °C). To determine the weight loss, the samples were removed from the NaOH solution at the given time, immersed in distilled water for 30 min, and freeze-dried. The residual weight (R.W.) was calculated as follows:

R.W. (%) = m/m_0_ × 100 (3)

where m is the weight of the sample at the time of measurement and m0 is the initial sample weight. The degradation was realized in triplicate.

### 4.6. Drug Loading and Release Studies

Hydrogel samples (30 mg) were immersed in a water solution of 20 mg/mL of LEV (with an additional 0.05% acetic acid) for 24 h in the dark and at room temperature to encapsulate the drug. The amount of loaded drug was obtained by measuring the concentration of the drug solution after loading. The LEV release experiments were carried out by immersing the loaded hydrogels in 10 mL of phosphate buffer solution—PBS (pH 7.4) at room temperature. Then, 2 mL of the release medium was extracted at specific time intervals and replaced with 2 mL of PBS.

The drug solutions were analyzed using a UV-Vis spectrophotometer (UV-1700 PharmaSpec Shimadzu), using standard quartz cuvettes of 1 cm^2^ base. The amount of drug was measured at a wavelength of 289 nm using a calibration curve for the concentration range between 0.003–0.008 mg/mL, (y = 87.206x, R^2^ = 0.994).

To identify the mechanism for the release of drugs, data obtained from in vitro drug release studies have been plotted as log (cumulative percentage of drug release) against log (time), using the Korsmeyer Peppas model of drug release kinetics. This can be represented using the following equation:

Mt/M∞ = Ktn(4)

where Mt/M∞ refers to the fraction of drug released at time t, Mt is the total amount released at time t, M∞ is the total amount of drug present in the hydrogels, t is release time in hours, K is kinetic constant, and n is the release exponent indicative of the release mechanism.

## Figures and Tables

**Table 1:** Synthesis parameters of CDCL-PEG hydrogels.

# Sample	Molar Ratio * CDCL:PEG–(NCO)_2_	Molar Ratio OH:NCO	Yields, %
C2	1:2	21:4	97
C4	1:4	21:8	97
C8	1:8	21:16	99
C12	1:12	21:24	96

* Molar ratios were calculated using the Mn results obtained from MALDI MS.

## Data Availability

Not applicable.

## Supplementary Materials

The following supporting information can be downloaded at: https://www.mdpi.com/article/10.3390/gels9090755/s1, Figure S1. SEM micrographs, cross-section view of the hydrogels at different magnifications; Figure S2. Amplitude sweep test performed for the CDCL-PEG hydrogel samples swollen in DMF; Figure S3. Frequency sweep test performed for the CDCL-PEG hydrogel samples swollen in DMF; Figure S4. Micrographs of C4 sample, section view, after (a) 1 day, (b) 3 days, (c) 5 days, and (d) 10 days of degradation; Figure S5. Surface micrographs of the C8 sample after (a) 1 day, (b) 3 days, (c) 5 days, and (d) after 10 days of degradation; Figure S6. Micrographs of C8 sample, section view, after (a) 1 day, (b) 3 days, (c) 5 days, and (d) 10 days of degradation; Figure S7. The drug release profiles of the hydrogels: (a) the levofloxacin released amounts and (b) the release efficiency after 24h; Figure S8. Korsmeyer-Peppas model plot for C2, C4, C8 and C12 samples; Table S1. Degradation data from TG and DTG curves; Table S2. The calculated time test parameters for hydrogels; Tabel S3. The limits of the linear viscoelastic range (ɣLVE) and ΔG for hydrogels; Table S4. DSC data for CDCL-PEG hydrogels; Table S5. The theoretical content of the hydrogels according to synthesis feed vs. residual weight after hydrolytic degradation; Table S6. Levofloxacin loading and release.
