# Supplementary material for: Polyurethane Degradable Hydrogels Based on Cyclodextrin-Oligocaprolactone Derivatives
Source: Gels. 2023 Sep 16;9(9):755. doi: 10.3390/gels9090755 (PMC10530861; doi:10.3390/gels9090755)
Supplement: Supplementary file 1 [file gels-09-00755-s001.zip › gels-2568049-supplementary.pdf]

Supplementary Materials: Polyurethane Degradable Hydrogels Based on Cyclodextrin-Oligocaprolactone Derivatives

Alexandra-Diana Diaconu [1], Corina-Lenuta Logigan [2], Catalina Anisoara Peptu [2], Constanta Ibanescu [2], Valeria Harabagiu [1] and Cristian Peptu [1,*]

[1] "Petru Poni" Institute of Macromolecular Chemistry, Aleea Grigore Ghica Voda 41A, 700487 Iasi, Romania; diaconu.diana@icmpp.ro (A.-D.D.); hvaleria@icmpp.ro (V.H.)
[2] Department of Natural and Synthetic Polymers, Faculty of Chemical Engineering and Environmental
Protection, "Gheorghe Asachi" Technical University of Iasi, 71, Prof. Dr. Docent Dimitrie Mangeron Street, 700050 Iasi, Romania; savincorina@yahoo.com (C.-L.L.); catipeptu@yahoo.co.uk (C.A.P.); cibanescu@tuiasi.ro (C.I.)
* Correspondence: cristian.peptu@icmpp.ro

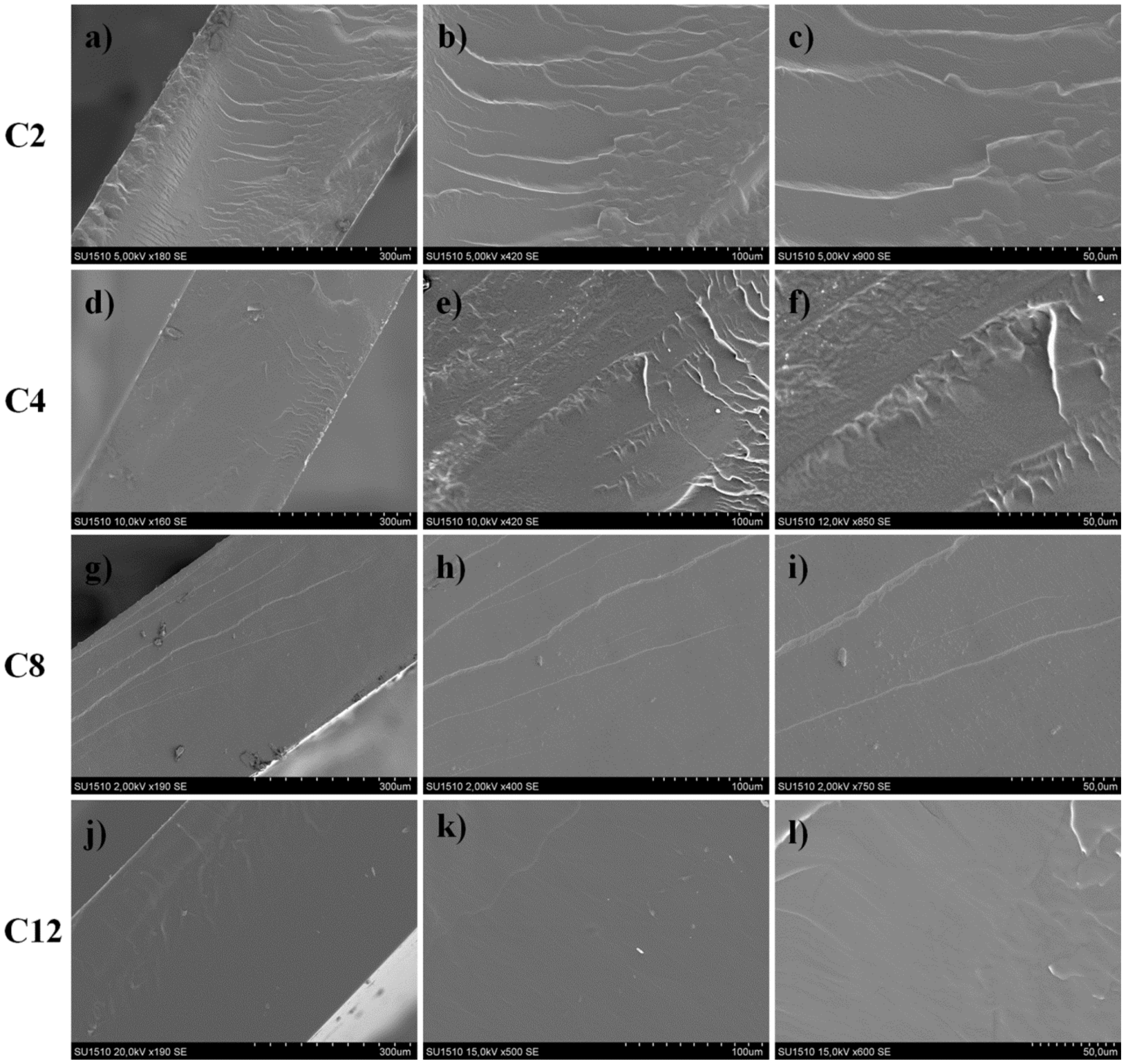

Figure S1. SEM micrographs, cross-section view of the hydrogels at different magnifications

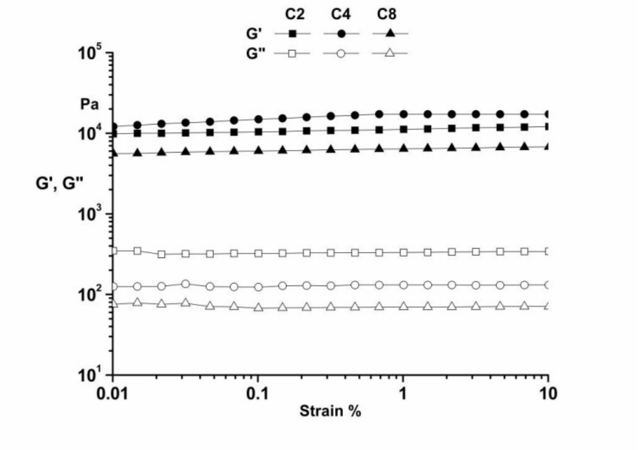

Figure S2. Amplitude sweep test performed for the CDCL-PEG hydrogel samples swollen in DMF

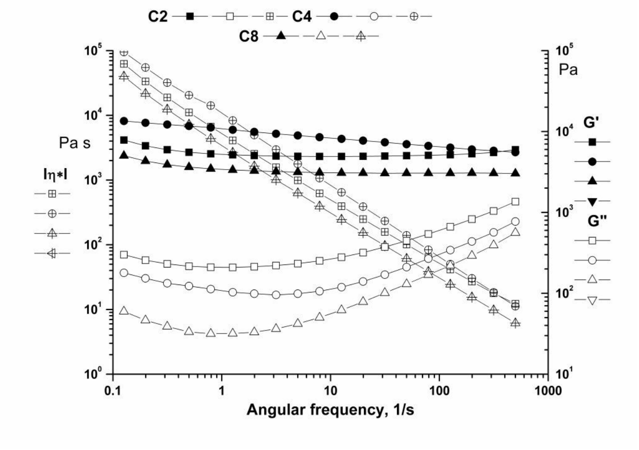

Figure S3. Frequency sweep test performed for the CDCL-PEG hydrogel samples swollen in DMF

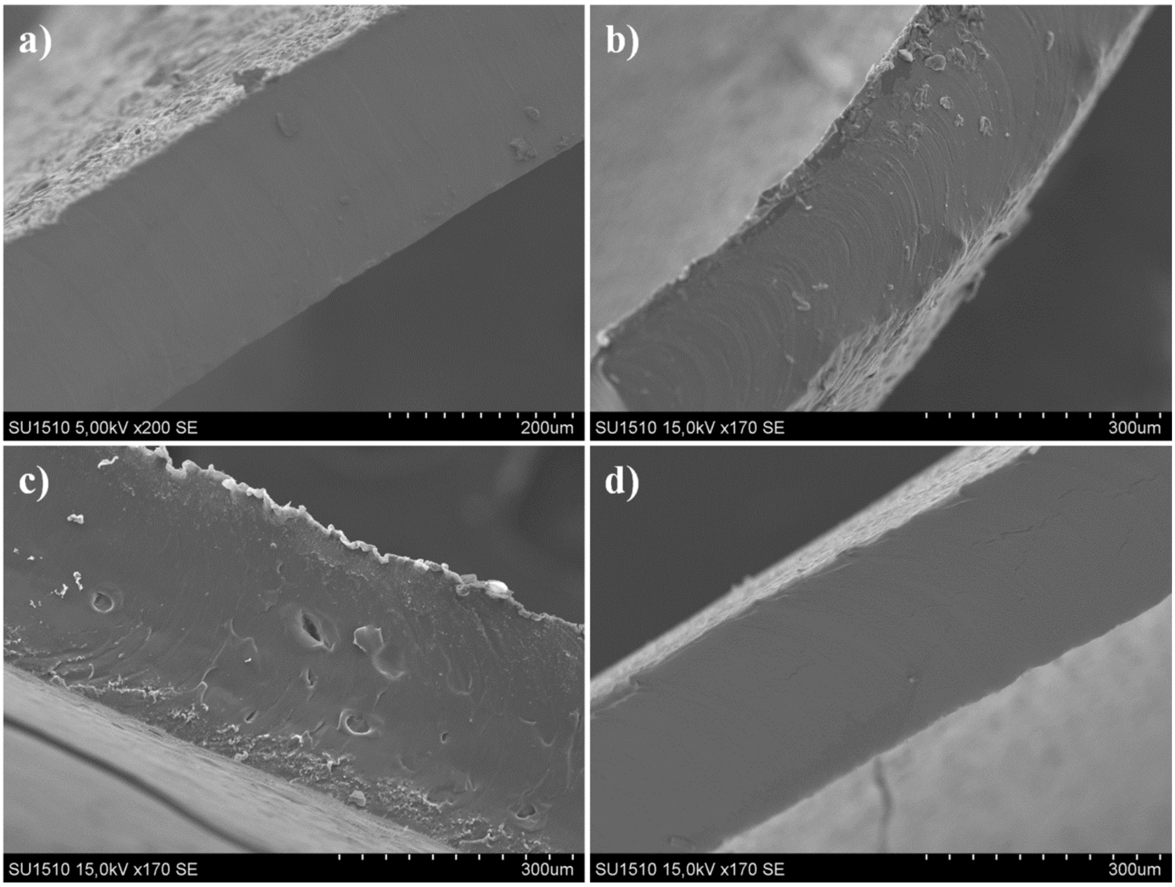

Figure S4. Micrographs of C4 sample, section view, after a) 1 day, b) 3 days, c) 5 days, and d) 10 days of degradation

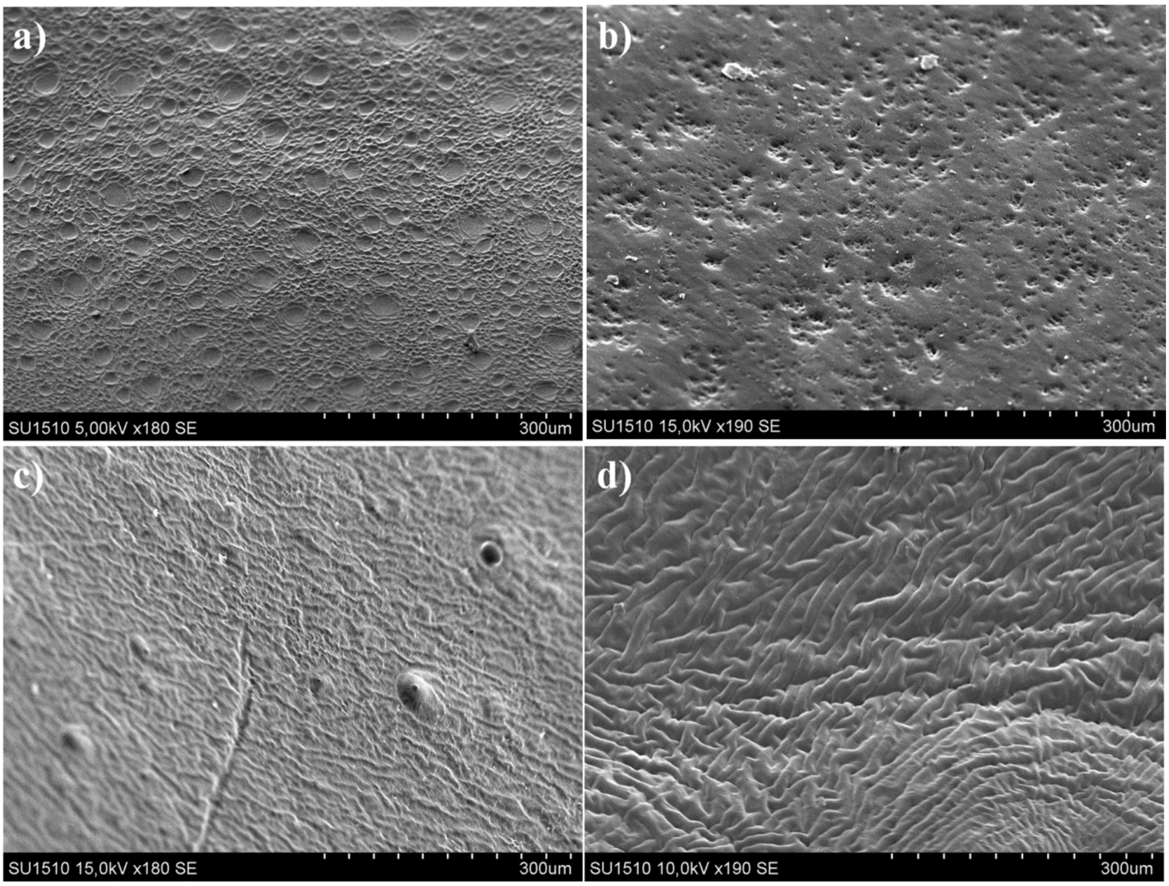

Figure S5. Surface micrographs of the C8 sample after a) 1 day, b) 3 days, c) 5 days, and d) after 10 days of degradation

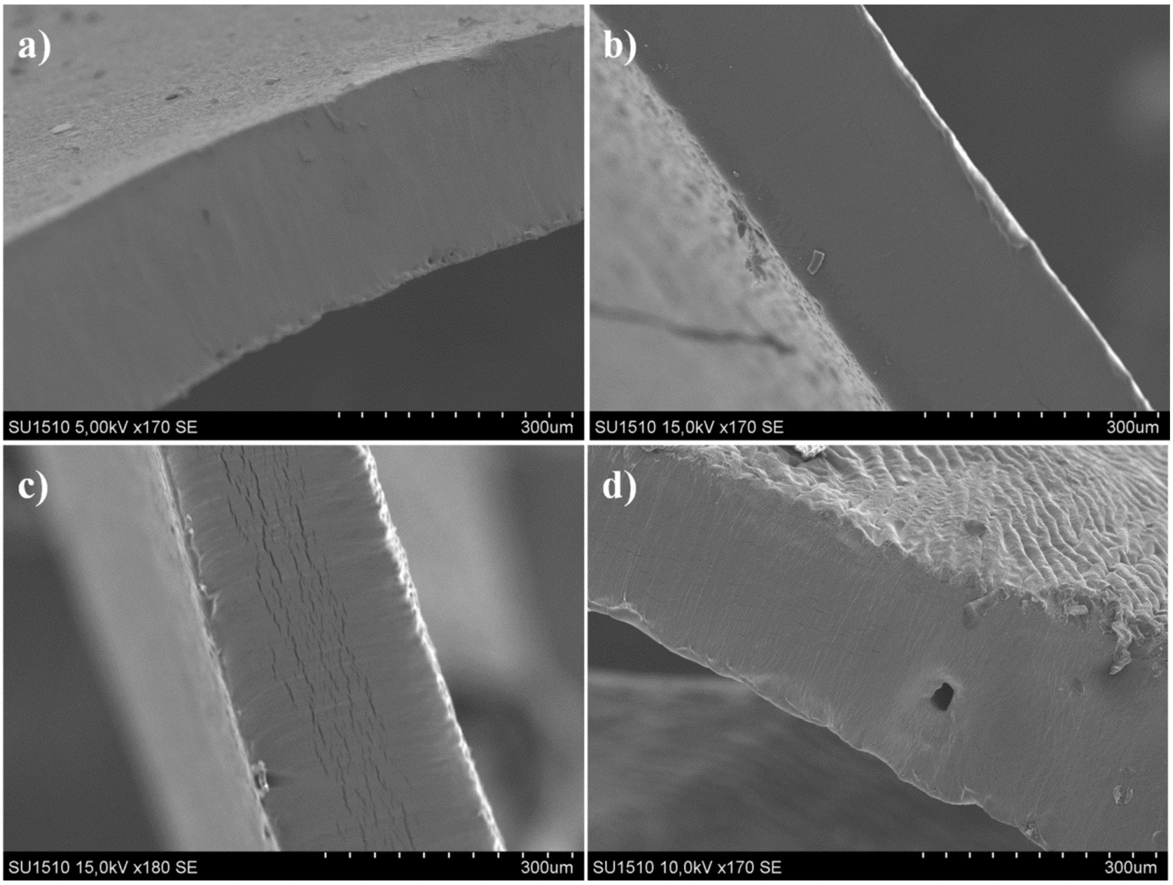

Figure S6. Micrographs of C8 sample, section view, after a) 1 day, b) 3 days, c) 5 days, and d) 10 days of degradation

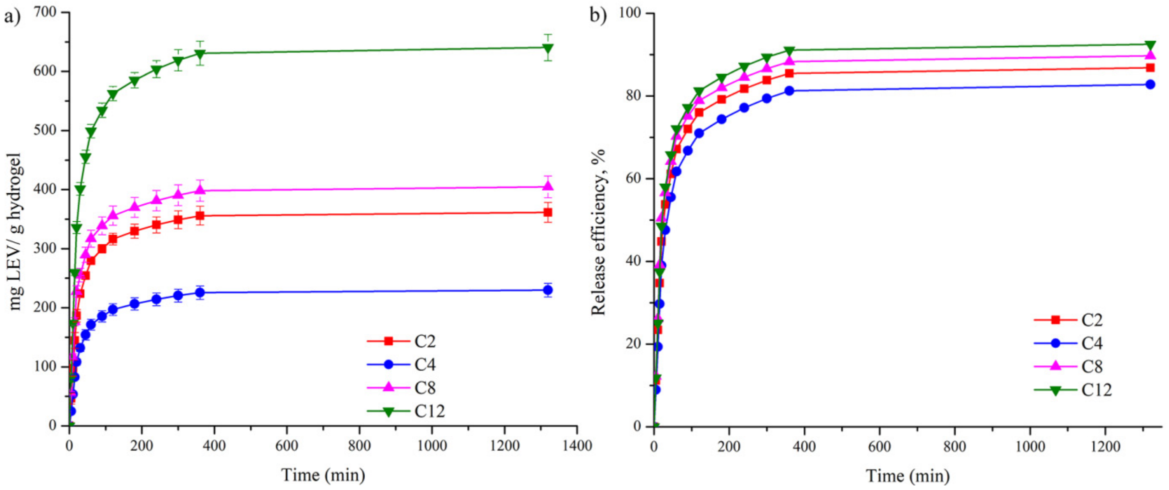

Figure S7. The drug release profiles of the hydrogels: a) the levofloxacin released amounts and b) the release efficiency after 24h

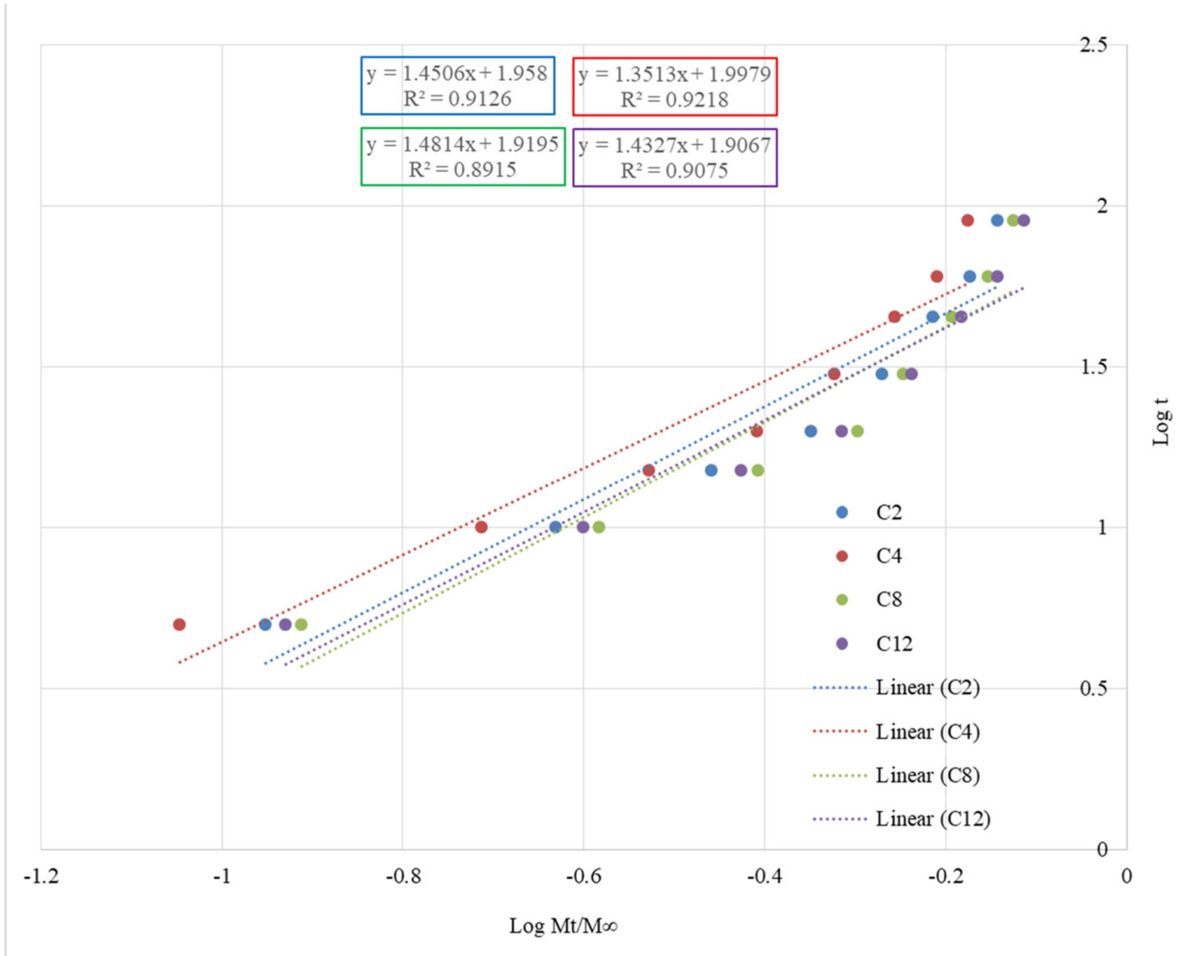

Figure S8. Korsmeyer-Peppas model plot for C2, C4, C8 and C12 samples

Table S1. Degradation data from TG and DTG curves

#Sample	Degradation range (°C)		Peak (°C)	weight loss (%)
**CDCL**	Step 1	264.84-311.55	304.19	11.063
	Step 2	314.25-420.09	361.07	90.821
**C2**	Step 1	306.73-348.66	332.24	8.253
	Step 2	348.66-393.29	369.76	29.143
	Step 3	395.61-459.23	425.12	93.09
**C4**	Step 1	300.55-343.16	336.53	7.8
	Step 2	343.79-379.72	355.19	21.507
	Step 3	381.62-468.58	424.97	96.19
**C8**	Step 1	303.3-343.83	334.2	8.125
	Step 2	343.84-375.42	353.13	20.44

	Step 3	376.4-456.52	419.47	97.413
**C12**	Step 1	304.69-343.85	320.95	9.802
	Step 2	344.41-372.01	360.08	20.268
	Step 3	375.84-456.71	420.96	98.093

Table S2. The calculated time test parameters for hydrogels

Sample	$t_{sol\text{-}gel}$ (min)	G′=G″ (Pa)	lη*l (Pa·s)
C2	53.85	2.645	0.3783
C4	46.61	5.079	0.7181
C8	32.03	9.924	1.403

Tabel S3. The limits of the linear viscoelastic range (γ_{LVE}) and ΔG for hydrogels

Sample	γ_{LVE} (%)	ΔG = G′-G″ (Pa)
C2	5	10706
C4	5	17075
C8	5	6282

Table S4. DSC data for CDCL-PEG hydrogels

#Sample	T_g (°C)	T_{cr} (°C)	T_{melt} (°C)
**C2**	-50.3	-	22.32
**C4**	-51.44	-19.77	27.91
**C8**	-52.97	-27.19	32.63
**C12**	-53.64	-29.18	34.96

Table S5. The theoretical content of the hydrogels according to synthesis feed *vs.* residual weight after hydrolytic degradation

# Sample	CDCL/ PEG-$(NCO)_2$ molar ratio	Content, % wt			R.W. %			
					day			
		PEG-IPDI	CD	CL	1	3	5	10
**C2**	1:2	75.31	17.28	7.41	62.56	0	0	0
**C4**	1:4	85.92	9.86	4.23	80.31	78.24	72.35	68.08
**C8**	1:8	92.42	5.30	2.27	87.06	84.76	83.01	78.42
**C12**	1:12	94.82	3.63	1.55	93.01	89.79	87.74	82.76

Table S6. Levofloxacin loading and release

	C2	C4	C8	C12
**LEV loaded amount, mg**	12.49	8.33	13.53	20.77
**LEV – released, mg/g**	361.5	229.8	404.7	640.6
**LEV-released, %**	86.9	82.7	89.7	92.5
